# Phenotypic Switching of Nonpeptidergic Cutaneous Sensory Neurons following Peripheral Nerve Injury

**DOI:** 10.1371/journal.pone.0028908

**Published:** 2011-12-21

**Authors:** Ting Wang, Derek C. Molliver, Xiaotang Jing, Erica S. Schwartz, Fu-Chia Yang, Omar Abdel Samad, Qiufu Ma, Brian M. Davis

**Affiliations:** 1 School of Medicine, University of Pittsburgh, Pittsburgh, Pennsylvania, United States of America; 2 Division of Gastroenterology, Hepatology and Nutrition, Department of Medicine, University of Pittsburgh, Pittsburgh, Pennsylvania, United States of America; 3 Center for Neuroscience, University of Pittsburgh, Pittsburgh, Pennsylvania, United States of America; 4 Department of Anesthesiology, University of Pittsburgh, Pittsburgh, Pennsylvania, United States of America; 5 Pittsburgh Center for Pain Research, University of Pittsburgh, Pittsburgh, Pennsylvania, United States of America; 6 Dana-Farber Cancer Institute and Department of Neurobiology, Harvard Medical School, Boston, Massachusetts, United States of America; 7 Center for Neuroscience and Regeneration Research, Yale School of Medicine, New Haven, Connecticut, United States of America; Universidade Federal do Rio de Janeiro, Brazil

## Abstract

In adult mammals, the phenotype of half of all pain-sensing (nociceptive) sensory neurons is tonically modulated by growth factors in the glial cell line-derived neurotrophic factor (GDNF) family that includes GDNF, artemin (ARTN) and neurturin (NRTN). Each family member binds a distinct GFRα family co-receptor, such that GDNF, NRTN and ARTN bind GFRα1, -α2, and -α3, respectively. Previous studies revealed transcriptional regulation of all three receptors in following axotomy, possibly in response to changes in growth factor availability. Here, we examined changes in the expression of GFRα1-3 in response to injury *in vivo* and *in vitro*. We found that after dissociation of adult sensory ganglia, up to 27% of neurons die within 4 days (d) in culture and this can be prevented by nerve growth factor (NGF), GDNF and ARTN, but not NRTN. Moreover, up-regulation of ATF3 (a marker of neuronal injury) *in vitro* could be prevented by NGF and ARTN, but not by GDNF or NRTN. The lack of NRTN efficacy was correlated with rapid and near-complete loss of GFRα2 immunoreactivity. By retrogradely-labeling cutaneous afferents *in vivo* prior to nerve cut, we demonstrated that GFRα2-positive neurons switch phenotype following injury and begin to express GFRα3 as well as the capsaicin receptor, transient receptor potential vanilloid 1(TRPV1), an important transducer of noxious stimuli. This switch was correlated with down-regulation of Runt-related transcription factor 1 (Runx1), a transcription factor that controls expression of GFRα2 and TRPV1 during development. These studies show that NRTN-responsive neurons are unique with respect to their plasticity and response to injury, and suggest that Runx1 plays an ongoing modulatory role in the adult.

## Introduction

GDNF family ligands (GFL) are neurotrophic factors that regulate the development and functional phenotype of peripheral sensory neurons in the dorsal root ganglia (DRG). The GFL receptor complex consists of Ret, a receptor tyrosine kinase, in combination with a member of the GFRα family of glycophosphatidylinositol-linked receptors (GFRα1-4). More recently, neural cell adhesion molecule (NCAM) and integrin have been implicated as potential co-receptors for GFL [1], [2], [3], [4]. Following peripheral nerve injury, successful regeneration requires a program of gene expression that includes changes in growth factor receptor expression [5], [6], [7]. *In vivo* studies of sensory neurons following peripheral axotomy have found increases in the percentage of neurons expressing GFRα1 and GFRα3 mRNA (the receptors for GDNF and ARTN, respectively) and a decrease in the percentage of GFRα2 (the receptor for NRTN) [8], [9].

It has been proposed that injury-induced alterations in peripheral growth factor expression lead to changes in GFRα expression in sensory neurons [8], the inference being that GFL can regulate the level of their cognate receptors. The majority of GFRα1 and α2 neurons are non-peptidergic (i.e., do not express the pro-inflammatory neuropeptides calcitonin gene-related peptide (CGRP) or substance P (SP)) C-fibers, whereas virtually all GFRα3-expressing neurons are peptidergic and 80% also express TrkA, the tyrosine kinase receptor for nerve growth factor (NGF) [10], [11], [12], [13], [14]. GFL protect against some of the pathological effects of nerve injury, including loss of neuropeptide expression and decreased conduction velocity [15]. In addition, both GDNF and NGF have been shown to inhibit expression of the transcription factor ATF3 [15], [16], which is normally induced in injured sensory neurons [17]. ATF3 is not only an effective marker of injured neurons, but also a driver of peripheral nerve regeneration [18].

Recent evidence indicates that, like GDNF and NGF, ARTN can reverse some effects of nerve injury [19]. However, unlike those factors, ARTN substantially improves functional recovery after dorsal root injury as well as peripheral nerve injury, and this recovery includes neurons that do not normally express the receptor GFRα3 [20].

The present study shows that NGF and ARTN regulate ATF3 expression and neuronal survival *in vitro*, whereas NRTN is ineffective. *In vivo*, we demonstrate that in some cells the loss of detectable GFRα2 is replaced by GFRα3 expression and that this may be regulated by Runx1, a transcription factor critical for differentiation of nociceptor subtypes. These changes have important functional consequences for nociceptive transduction in that neurons down-regulating GFRα2 subsequently begin to express *de novo* functional TRPV1 channels. These results indicate that injury-evoked changes in GFRα receptor expression alter the efficacy of the GDNF family members, and may explain the unexpected gain of function for ARTN and the loss of function for NRTN during sensory neuron regeneration.

## Results

### Distribution of GFRα1-3-expressing sensory neurons in vivo and 24 h *in vitro*

**Figure 1:**
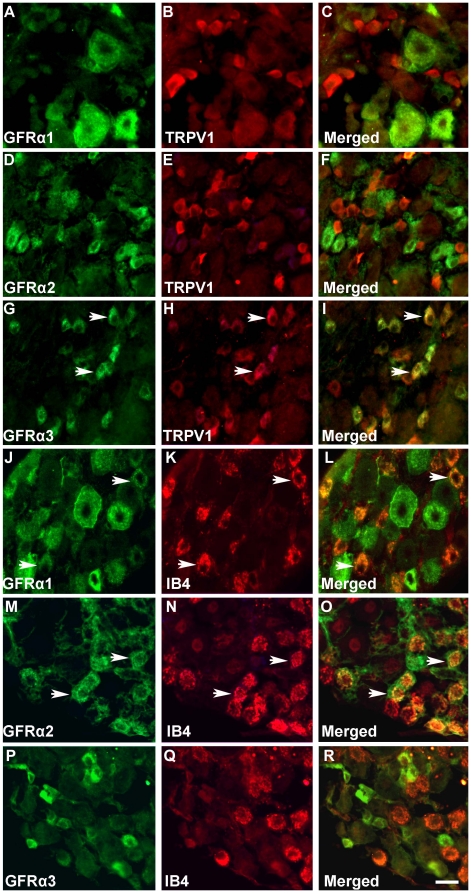
Immunolabeling for GFRα1, 2 or 3 and TRPV1 and IB_4_ in L4 DRG. Most GFRα1- and GFRα2-positive neurons did not express TRPV1 (***A–F***). GFRα3-positive neurons expressed TRPV1 (arrow = double labeled cells) (***G–I***). GFRα2-positive (***M–O***) and small diameter GFRα1-positive neurons (***J–L***) bind IB_4_ (arrows). Scale bar = 50 µm.

Initial experiments were designed to determine the proportion of neurons expressing GFRα1-3 protein *in situ* (i.e., tissue sections of DRG) and in dissociated neurons, to elucidate the extent to which the phenotype of cultured neurons was representative of the *in vivo* condition. Using recently validated GFRα1-3 antibodies [13], [21], we performed immunohistochemical staining on lumbar 4 (L4) DRG. Immunoreactivity for TRPV1 was also examined because it is expressed in 95% of GFRα3 neurons, but in only a minority of neurons labeled by the plant lectin IB_4_, that is extensively colocalized with GFRα1 and α2 [15]. A large number of cells immunoreactive for GFRα3 and TRPV1 were observed in L4 DRG of naïve mice (**Fig. 1** ***G, H, I***), whereas IB_4_ was primarily localized in GFRα2-positive (**Fig. 1** ***M, N, O***) and small-diameter GFRα1-positive neurons (**Fig. 1** ***J, K, L***), confirming previous studies [8], [15], [22].

**Figure 2:**
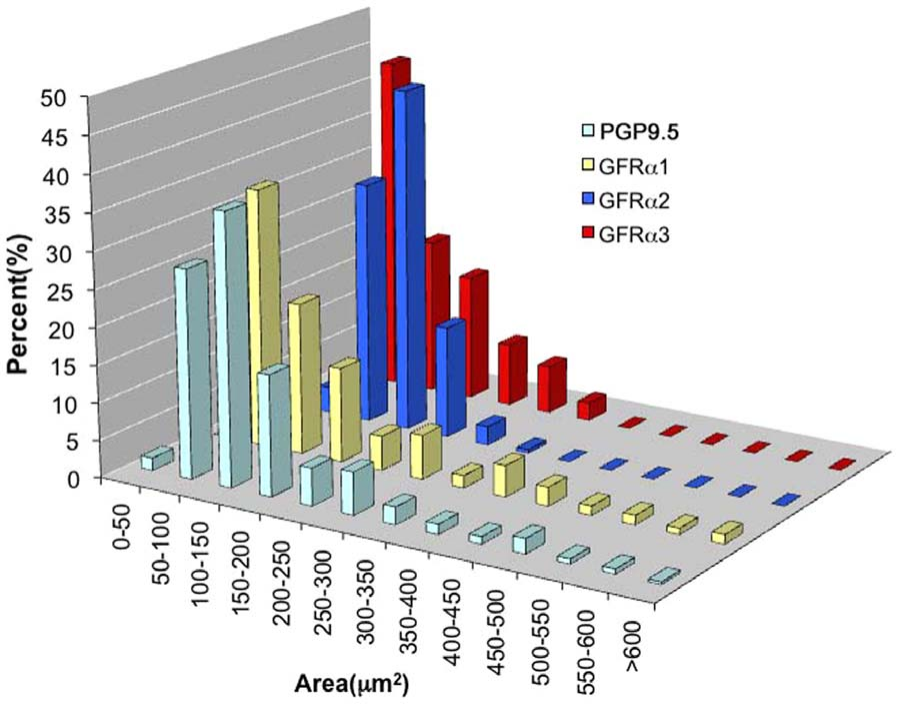
Cell size distribution of PGP9.5-, GFRα1-, GFRα2- and GFRα3-positive neurons in L4-5 DRG. Neurons were sorted by their cell area and the percent of neurons within each 50 µm bin were plotted. PGP9.5 staining was used to obtain the distribution for all sensory neurons. Note that GFRα1-immunoreactivity was expressed in both small and large sized neurons. Most GFRα2-positive neurons had areas between 100–250 µm^2^ whereas over 70% of GFRα3-positive neurons had areas of <200 µm^2^.

**Figure 2** shows the size distribution of GFRα1-, GFRα2- and GFRα3- positive somata in L4 DRG. The size distribution of each GFRα population was diverse; GFRα1-staining was seen in neurons with both the largest and smallest somata, whereas most GFRα2-positive neurons had somata with areas between 100–200 µm^2^. The majority of GFRα3-positive neurons had somata with areas below 150 µm^2^.

**Table 1** shows the percentage of mouse neurons that express GFRα1-, GFRα2- or GFRα3- immunoreactivity *in situ an*d after 1 d in culture (the time point typically used for acute physiological analysis of dissociated sensory neurons). Of all neurons identified using NeuN in intact ganglia, 33.5±1.9% expressed GFRα1-immunostaining, whereas 33.3±2.6% expressed GFRα2 and 24.8±0.8% expressed GFRα3, similar to what has been reported in rat and mouse [8], [14]. After 1 d in culture the percentages were 15.8±1.0, 2.1±0.6, and 43.9±2.9% for GFRα1, GFRα2, GFRα3, respectively. The decreases in both GFRα1- and α2- positive cells were statistically significant, as was the increase in GFRα3 (all *p values*<0.05; t-test).

**Table 1:** Percentage of neurons expressing GFRα1-, GFRα2-, GFRα3- and TRPV1- immunoreactivity in DRG (*in vivo*) and 1 d after culturing (*in vitro*).

	GFRα1	GFRα2	GFRα3	TRPV1	IB_4_
***in vivo*** ** (%)**	33.5±1.9	33.3±2.6	24.8±0.8	30.3±1.5	28.3±0.8
***in vitro*** ** (%)(24 h)**	15.8±1.0*	2.1±0.6%*	43.9±2.9*	36.3±0.6*	43.6±1.4*

*In vivo*, the percentage of neurons expressing GFRα1-3 was similar. *In vitro*, GFRα2-positive cells were rarely seen 1 d after plating. The percentage of GFRα1-positive cells was decreased whereas the percentage of GFRα3-positive cells was increased. A small increase in the percentage of TRPV1-positive and IB_4_-binding neurons also occurred (*p<0.05, t-test).

### Extent of cell death *in vitro* and effects of growth factors

To determine whether the decrease in GFRα1 and GFRα2 expression was due to selective loss of specific cell populations after dissociation, we counted the number of neurons at 6 hours (h) (the time point at which all cells that survived the dissociation process had attached to the coverslip), 1 d and 4 d after plating. Cells were grown on coverslips containing a numbered grid so that the same cells could be followed throughout the experiment. As shown in **Table 2**, in the control condition (no exogenous growth factor) 85.31±0.69% and 73.22±3.27% of neurons survived at 1 d and 4 d after plating, respectively. The minimal loss of cells at 1 d indicates that the decrease in the percent of cells expressing GFRα1 or GFRα2 was not only due to selective cell death, but was probably due to down-regulation of receptor expression. NGF (50 ng/ml), GDNF (50 ng/ml) or ARTN (20 ng/ml) significantly increased neuronal survival at both 1 and 4 d, whereas addition of NRTN (50 ng/ml) did not prevent cell loss.

**Table 2:** Sensory neuron survival *in vitro*.

	Control	NGF	GDNF	Artemin	NRTN
**6 h (%)**	100	100	100	100	100
**1 d (%)**	85.31±0.69	96.24±1.13*	92.38±2.06*	94.12±2.09*	81.95±5.23
**4 d (%)**	73.22±3.27	89.53±1.64*	87.71±1.80*	88.48±1.94*	76.16±4.43

The number of NeuN-positive neurons 6 h after plating was designated as 100%. Without growth factors (Control), approximately 15% of neurons died 1 d after plating and 27% died by 4 d. Application of NGF, GDNF and ARTN significantly increased neuron survival although NRTN was ineffective.

*p<0.05; Two way ANOVA, Dunnett's post-hoc test.

### Expression of ATF3 is suppressed by NGF and ARTN but not by NRTN

**Figure 3:**
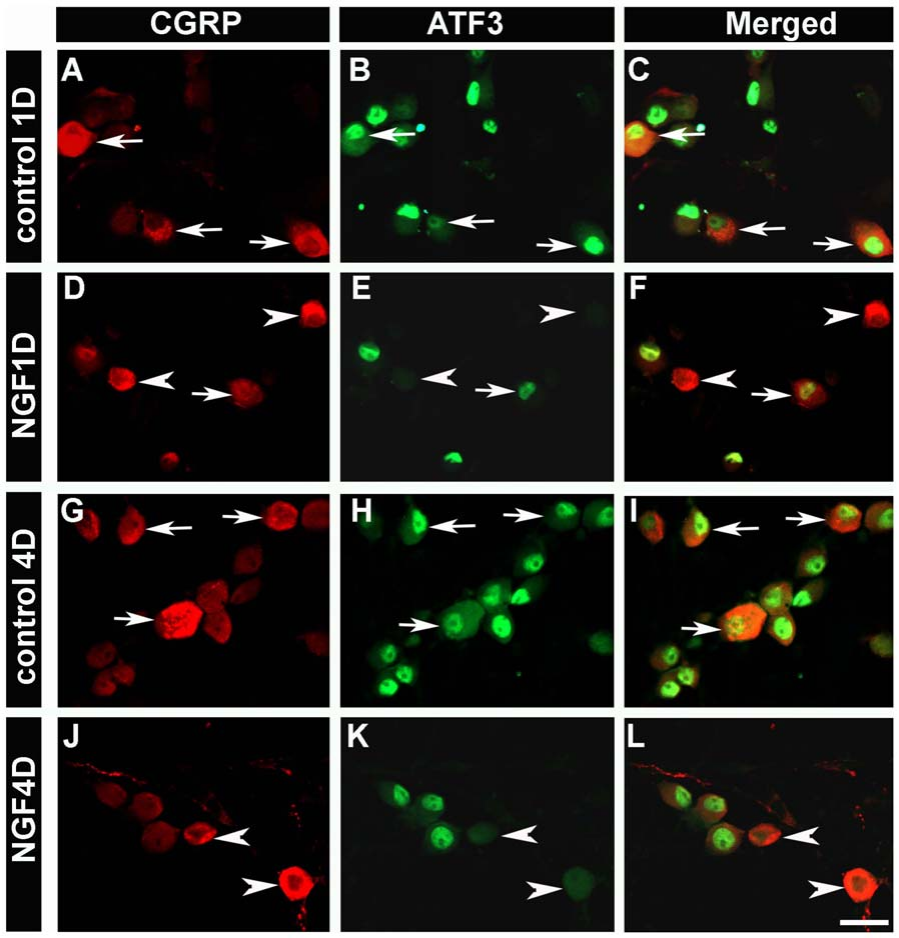
*In vitro* application of NGF decreased ATF3 expression in CGRP-positive neurons. In control conditions (no growth factor), more than 90% of CGRP-positive neurons expressed ATF3 at 1 d (92.9±0.3%) (***A–C***; arrows) and 4 d (93.4±0.4%) (***G–I***; arrows) after plating. In NGF-treated cultures, the percentage of CGRP-positive neurons that expressed ATF3 was significantly decreased at 1 d (3.8±0.3%) (***D–F***; arrowheads) and 4 d (3.6±0.3%) (***J–L***; arrowheads). Scale bar = 50 µm. Two way ANOVA, *p*<0.01.

**Figure 4:**
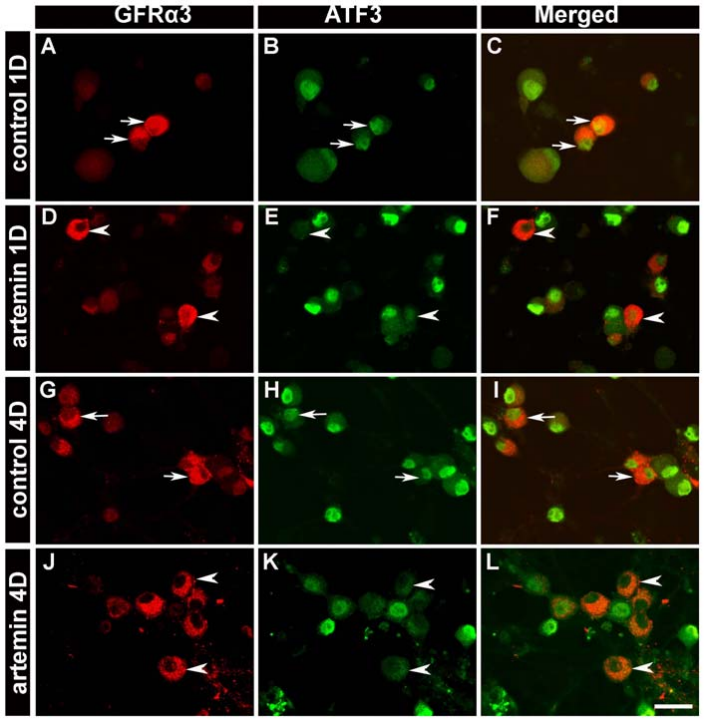
*In vitro* application of ARTN decreased ATF3 expression in GFRα3-positive neurons. In control conditions, most GFRα3-positive neurons expressed ATF3 at 1 d (96.7±0.5%) (***A–C***, arrows) and 4 d (97.2±0.4%) (***G–I***; arrows) after plating. Addition of ARTN significantly decreased the percentage of GFRα3-positive neurons that expressed ATF3 at 1 d (8.7±0.3%) (***D–F***; arrowheads) and 4 d (8.9±0.4%) (***J–L***; arrowheads). Scale bar = 50 µm. Two way ANOVA, *p*<0.01.

Because cell death did not appear to account for the dramatic loss in the percent of GFRα2-expressing neurons, we examined whether there were overt differences in the pattern of gene expression in different neuronal populations following dissociation. ATF3 is a transcription factor that has been shown previously to be expressed in the vast majority of axotomized afferents [17], and is thought to regulate the regeneration program [18] under the control of growth factors ([16], [23] (but see [24]). To test how growth factors regulate ATF3 expression in different primary afferent populations after dissociation *in vitro*, we applied NGF (50 ng/ml), ARTN (20 ng/ml) or NRTN (50 ng/ml) at the time of plating. 1 d after plating, 92% of DRG neurons (identified via NeuN expression) expressed ATF3 immunoreactivity and this widespread distribution was observed for up to 7 d in culture without exogenous growth factors. To determine the impact of NGF on ATF3 expression, cells were stained with an antibody to CGRP, which has been shown previously to be expressed in 96% of neurons expressing TrkA (the specific NGF receptor) [25]. Addition of NGF to the culture media eliminated ATF3 expression in 96% of CGRP-immunoreactive neurons 1 and 4 d after culture (**Fig. 3**). Similarly, application of ARTN eliminated ATF3 expression in 91% of GFRα3-immunoreactive neurons 1 and 4 d in culture (**Fig. 4**).

**Figure 5:**
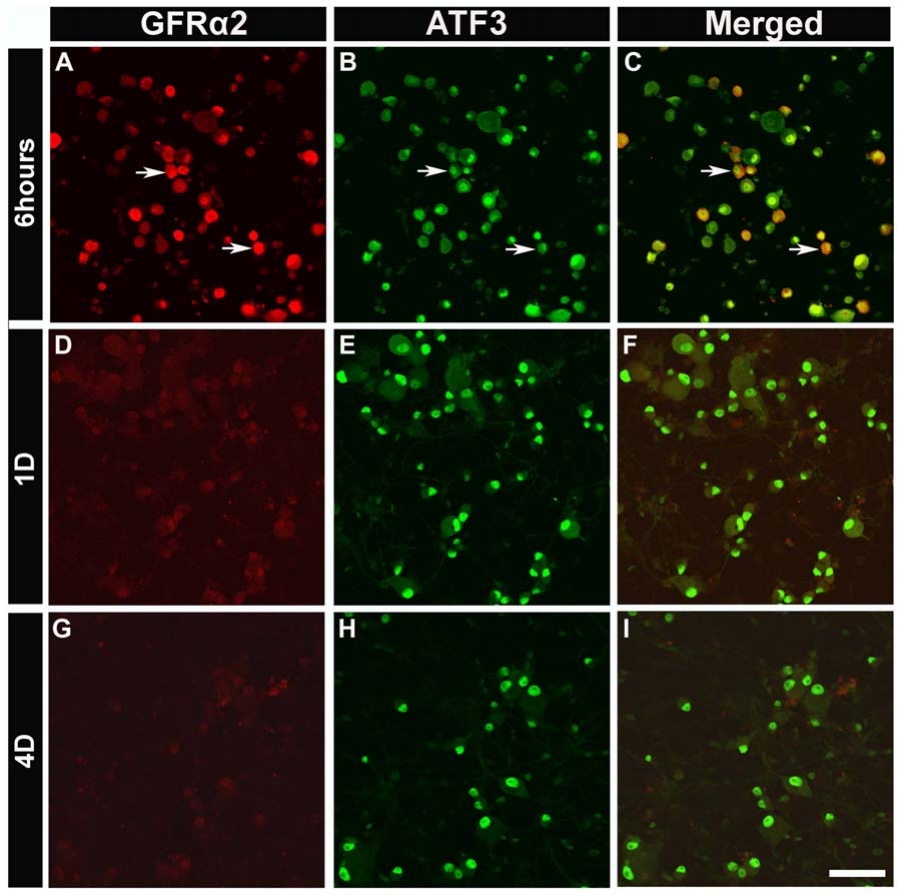
The percentage of GFRα2-positive neurons decreased after plating. 6 h after plating, 32% of neurons expressed GFRα2 (***A***, arrow) and most GFRα2-positive neurons expressed ATF3 (***B & C***; arrows). 1 d and 4 d after plating, the expression of GFRα2 could not be detected (***D*** **,** ***F***) and this was not reversed by application of NRTN or GDNF. Scale bar = 100 µm.

GFRα2 immunoreactivity could still be detected 6 h after plating, and at this time point, ATF3 expression was seen in virtually all GFRα2-expressing neurons (**Fig. 5** ***A–C***). However, GFRα2 expression was dramatically decreased after 1 d in culture and was virtually absent by day 4 (**Fig. 5** ***D–I***). Because the percentage of both GFRα1- and GFRα2-positive cells was decreased *in vitro* (**Table 1**), we examined the effect of NRTN or GDNF on all sensory neurons identified by NeuN staining. NRTN or GDNF application had no effect on the percentage of cells expressing ATF3: 92% of all NeuN-positive cells expressed ATF3 in the presence of either growth factor. This was true even at 6 h after plating: over 95% of all neurons expressed ATF3 in the presence or absence of GDNF or NRTN (data not shown).

We repeated these studies using IB_4_ labeling as a phenotypic marker specifically for GFRα1 and GFRα2 neuronal populations. In neurons from mice that lack GFRα2, the percentage of IB_4_-positive cells is identical to that in cultures established from wild type mice [26], indicating that IB_4_ binding is independent of GFRα2 expression. Addition of NRTN, GDNF, or a combination of NRTN and GDNF was not able to suppress the expression of ATF3 in IB_4_-binding neurons: 84.6±1.4% of IB_4_-positive neurons expressed ATF3 after 1 d in culture and this number was unchanged following addition of NRTN or GDNF (86.9±1.4% and 86.4±2.5%, respectively). After 4 d in culture 78.2±1.5% of IB_4_-positive cells were immunoreactive for ATF3, whereas 77.9±1.6% and 79.0±2.3% were ATF3-positive despite the addition of NRTN or GDNF. Addition of a combination of both growth factors also had no effect on ATF3 expression at 1 d and 4 d (86.0±2.1% and 80.0±1.6% of IB_4_-positive neurons were ATF3-positive at 1 and 4 d, respectively, after addition of both GDNF and NRTN combined) (**Table 3**).

**Table 3:** Application of NRTN and/or GDNF does not suppress ATF3 expression in IB_4_-binding neurons.

	Control	NRTN	GDNF	GDNF+NRTN
1 d(%)	84.6±1.4	86.9±1.4	86.4±2.5	86.0±2.1
4 d(%)	78.2±1.5	77.9±1.6	79.0±2.3	80.0±1.6

1 d and 4 d after culturing, cells were stained with IB_4_ and ATF3. The percentage of IB_4_-binding neurons that expressed ATF3 is shown. Compared with control, application of NRTN, GDNF, or both, did not decrease ATF3 expression in IB_4_-binding neurons. Two-way ANOVA.

To confirm whether the failure of NRTN to suppress ATF3 expression was a consequence of the loss of GFRα2, we added exogenous soluble GFRα2 with NRTN to dissociated neurons. This experiment was based on a previous study by Ernfors and colleagues, who manipulated neurite outgrowth in sensory neuron cultures using soluble GFRα1, indicating that addition of soluble GFRα1 plus GDNF was able to engage downstream signaling pathways [27]. We used this paradigm to determine if NRTN, in the presence of soluble GFRα2, could block ATF3 expression. GFRα2 (200 ng/ml) and NRTN (100 ng/ml) were added to cultures at the same time that cells were plated. One day later, 86.7±2.1% IB_4_-positive neurons expressed ATF3 (similar results were observed with 100 or 400 ng/ml soluble GFRα2; data not shown). This percent was not different from the result without GFRα2 (**Table 3**), indicating that NRTN does not regulate ATF3 expression even in the presence of GFRα2.

### *In vivo*, axotomized GFRα2-expressing neurons down-regulate GFRα2 and express GFRα3

**Figure 6:**
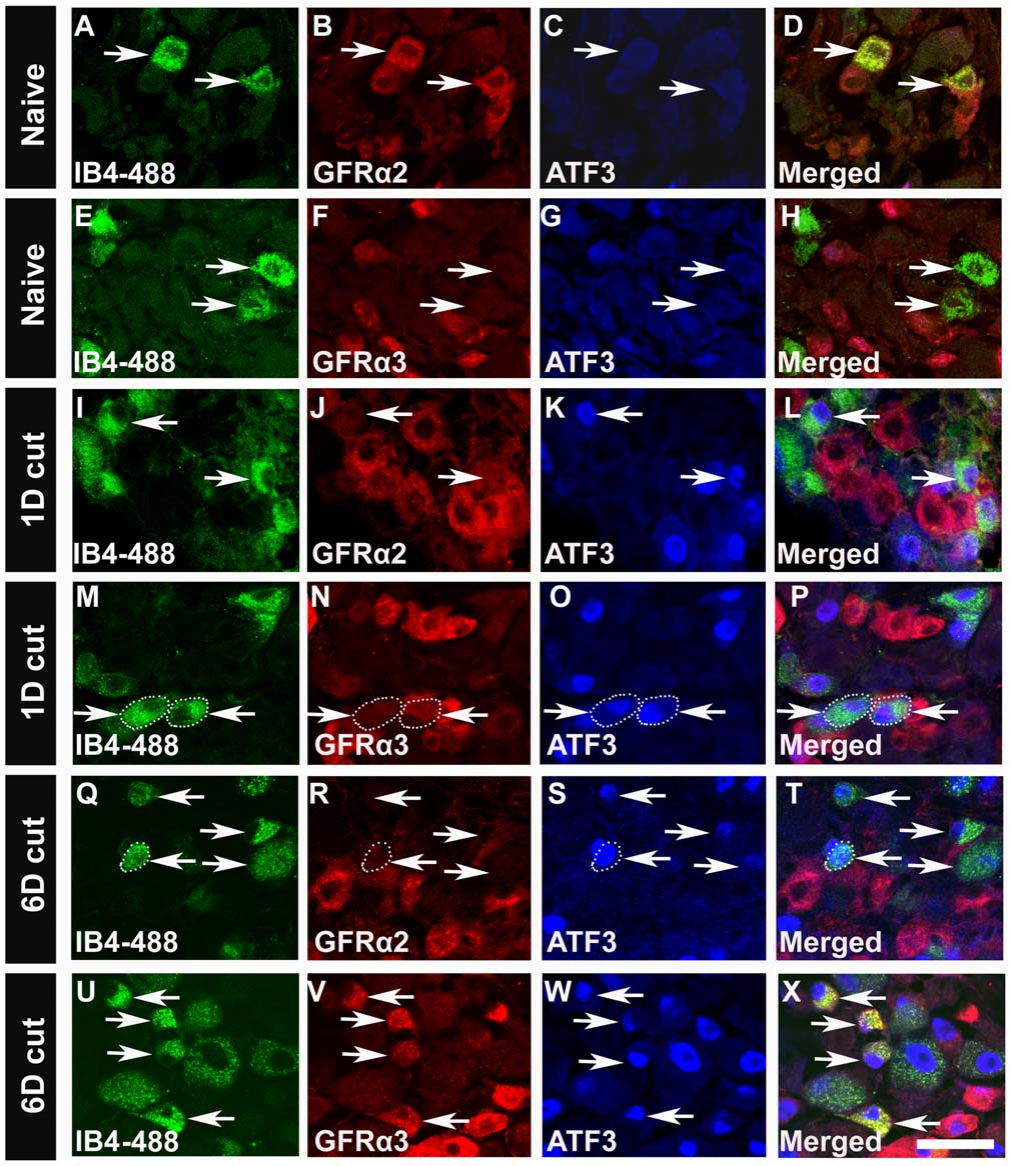
After saphenous nerve injury GFRα2-positive neurons down-regulated GFRα2 and expressed GFRα3. In naïve mice, IB_4_-488-back-labeled neurons were immunopositive for GFRα2 (***A*** **,** ***B*** **,** ***D*** **;** arrows), but not GFRα3 (***E*** **,** ***F*** **,** ***H*** **;** arrows). No ATF3 expression was seen following IB_4_-488 injections in naïve mice (***C*** **,** ***G*** **;** arrows). One d after saphenous nerve lesion, IB_4_-488-back-labeled neurons expressed ATF3 (***I*** **,** ***K*** **,** ***M*** **,** ***O*** **;** arrows), however, GFRα2 expression in IB_4_-back-labeled neurons was decreased (***I*** **,** ***J*** **;** arrows). GFRα3 expression was not seen in IB_4_-488-labeled neurons 1 d post-transection (***M*** **,** ***N*** **,** ***P*** **;** arrows). 6 d after saphenous nerve lesion, IB_4_-488-back-labeled neurons expressed ATF3 (***Q*** **,** ***S*** **,** ***U*** **,** ***W*** **;** arrows) and GFRα3 (***U*** **,** ***V*** **,** ***X*** arrows), but not GFRα2 (***Q*** **,** ***R*** **,** ***T*** **;** arrows). Scale bar = 50 µm.

Previous studies in rat indicated a near doubling in the number of DRG neurons expressing mRNA for GFRα1 and GFRα3 following axotomy *in vivo*, such that over 66% of sciatic afferents expressed GFRα1 and 66% expressed GFRα3 by day 14 post-nerve cut [8]. Because the number of neurons expressing one or both of these receptors exceeds 100%, this indicates that some cells that did not previously express GFR -α1 or -α3, must now be expressing these receptors. The observed down-regulation of GFRα2 protein reported here and previously [8] makes these cells likely candidates for switching receptor phenotype. To examine the phenotype of GFRα2-positive neurons after peripheral nerve injury *in vivo*, fluorescently-tagged IB_4_ (IB_4_-488), that is selectively taken up by IB_4_-binding neurons, was injected subcutaneously into the dorsal medial portion of the hindpaw prior to nerve transection. When IB_4_-488 is injected into the hindpaw, it is retrogradely transported through the saphenous nerve to cutaneous somata in the L2-3 DRG, where it labels both GFRα1 and GFRα2-expressing neurons [15]. Three days after injection, IB_4_-488 labeled a large number of neurons expressing GFRα2 (**Fig. 6 *A–D***). No cells expressing GFRα3 were co-labeled with IB_4_-488 (**Fig. 6 *E–H***). In contrast, one day following saphenous nerve axotomy (4 d after IB_4_-488 skin injection), ATF3-immunoreactivity was seen in neurons retrogradely labeled with IB_4_ (**Fig. 6 *I–P***). By this time, the GFRα2 expression had already decreased to such an extent in injured neurons that it was difficult to find neurons that were GFRα2-positive and ATF3-positive, or GFRα2-positive cells that were back-labeled with IB_4_-488 (**Fig. 6 *I–L***). By day 6 post-axotomy, 38.92±2.81% of IB_4_-488 back-labeled neurons expressed GFRα3 (n = 4) (**Fig. 6 *U–X***) indicating that a significant proportion of neurons that previously expressed GFRα1 or GFRα2, now expressed GFRα3.

To determine how gene expression was changing at the single cell level, DRG cultures containing IB_4_-back-labeled neurons, 1 and 6 d post-axotomy, were used to harvest individual cells for mRNA analysis. For these studies, 20 IB_4_-back-labeled neurons per mouse were analyzed by RT-PCR. For each treatment (naïve (non-axotomized), 1 d post-axotomy, 6 d post-axotomy), 3 mice were used (total of 60 cells/treatment). Consistent with the immunostaining results, all IB_4_-labeled neurons expressed GFRα2 mRNA in naïve DRG (**Table 4**), whereas GFRα1 and GFRα3 mRNA was identified in 8.0±0.5 (40%) and 5.7±0.3 (29%) cells out of 20 IB_4_-back-labeled neurons/mouse, respectively. 1 and 6 d after axotomy, all IB_4_-labeled neurons expressed ATF3 mRNA and the number of neurons expressing GFRα2 mRNA decreased to <1/mouse (**Table 4**). No change was seen in the other genes examined 1 d post axotomy, but by 6 d, the number of IB_4_-labeled neurons expressing GFRα1 and GFRα3 increased significantly. These changes in transcription mirror the changes in immunhistochemical staining (**Figure 6**) and confirm previous studies in rat [8]. However, GFRα1 mRNA distribution was stable at 1 d post-axotomy, whereas we saw a decrease in staining for GFRα1 after 1 d in culture (**Table 1**). This difference could reflect that in the immunohistochemical studies we analyzed all cells, not just IB4-back-labeled cutaneous afferents. This difference could also reflect a difference between protein and mRNA expression.

**Table 4:** The number of neurons expresses GFRα1-3, TRPV1 and ATF3 mRNA out of 20 IB_4_-labeled neurons in DRG after axotomy.

	GFRα1	GFRα2	GFRα3	TRPV1	ATF3
**Naïve**	8.0±0.5 (40%)	20 (100%)	5.7±0.3 (29%)	5.3±0.3 (27%)	0 (0%)
**1 d**	8.3±0.3 (42%)	0.3±0.3 (.02%)	6.0±0.5 (30%)	6.0±0.5 (30%)	20* (100%)
**6 d**	16.3±0.3* (82%)	0.3±0.3* (.02%)	15.3±0.5 (77%)	15.7±0.3* (79%)	20* (100%)

In naïve (non-axotomized) mice, all IB_4_-back-labeled neurons expressed GFRα2 mRNA. However, GFRα2 mRNA expression was rarely detected 1 and 6 d post-axotomy. The number of IB_4_-back-labeled neurons expressing GFRα1, GFRα3 and TRPV1 mRNA increased significantly 6 d post-axotomy. ATF3 mRNA expression was detected in all IB_4_-back-labeled neurons 1 d after nerve transection and remained at this level for at least 6 d. (n = 3, *p<0.05, t-test).

### Injured cutaneous afferents upregulate TRPV1

**Figure 7:**
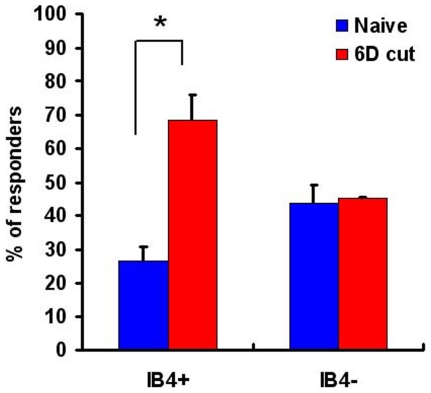
The percentage of IB_4_-488 labeled neurons responding to capsaicin was increased 6 d after nerve axotomy. In naïve mice, 26.5±4.3% of IB_4_-488 back-labeled neurons (IB_4_+) responded to capsaicin. The percentage increased to 68.5±7.5% 6 d after axotomy. In neurons that were not labeled by IB_4_-488 (IB_4_-), the percentage of capsaicin responders was not different before and after axotomy. Fisher's exact test, *p<0.01.

As shown in **Figure 6**, most IB_4_-488 back-labeled neurons from naïve mice expressed GFRα2. The single-cell RT-PCR data indicated that 27% of these express TRPV1 mRNA (**Table 4**). Six days after saphenous nerve axotomy, 77% of IB_4_-488 back-labeled neurons expressed GFRα3 (**Table 4**). Because most GFRα3-positive neurons express TRPV1 [13]–[14], we performed Ca^2+^ imaging to determine the proportion of IB_4_-488 back-labeled neurons responding to capsaicin in naïve and axotomized mice. In naïve mice (n = 3), 26.5±4.3% of IB_4_-488 back-labeled neurons responded to capsaicin (1 µM) (similar to previous reports [28]). However, by 6 d after saphenous nerve axotomy (n = 3), this percentage had more than doubled (68.5±7.5% (*p*<0.01; Fisher's exact test)). This is slightly lower than the percentage of IB_4_-488-back-labeled neurons that expressed TRPV1 mRNA (78.3%; **Table 4**). Again, this might reflect a difference between protein and mRNA and/or a difference in the sensitivity of the two techniques (Ca^2+^ imaging and PCR). No change occurred in the percentage of capsaicin responders that were not back-labeled with IB_4_-488 (capsaicin responses in naïve IB_4_-488-negative neurons = 43.6±5.5%: capsaicin responses in 6 d axotomized IB_4_-488-negative neurons = 45.1±0.3%) (**Fig. 7**). These data suggest that most IB_4_-488 back-labeled neurons were axotomized and that some of these neurons acquired TRPV1 function *de novo*, following injury.

### Changes in GFRα2 and Runx1 mRNA after axotomy

**Figure 8:**
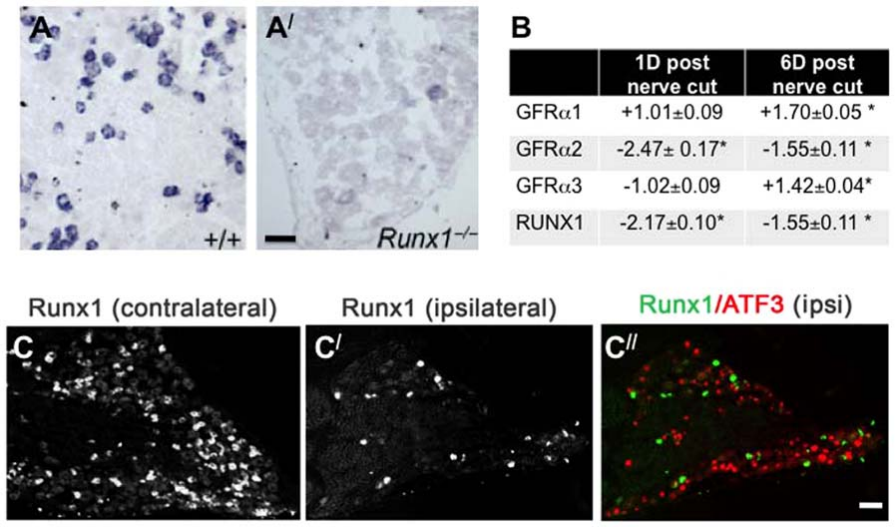
GFRα2 expression was dramatically decreased in Runx1 knockout mice. ISH for GFRα2 in WT (***A***) and Runx1 knock out (***A^/^***) mouse DRG showed significant reduction of GFRα2 in Runx1^−/−^ ganglia. **B**) Fold change of GFRα1-3 and Runx1 mRNA following sciatic nerve transection. GFRα2 and Runx1 decreased concurrently. GFRα1 and GFRα3 increased at 6 d post transection confirming previous rat ISH studies. *p<0.05; t-test. **C**) Five days after sciatic nerve axotomy, Runx1 expression decreased in ipsilateral DRG (***C^/^***) relative to contralateral DRG (***C***). Runx1 expression (green) was absent in ATF3-positive neurons in ipsilateral DRG (***C^//^***). Scale bar = 50 µm.

As noted above, previous studies in rat DRG showed changes in mRNA expression for GFRα1-3 14 d following sciatic nerve transection [8]. We confirmed these changes in mouse 6 d following saphenous nerve transection using single cell RT-PCR (**Table 4**) and on the whole DRG level following sciatic nerve transection (**Fig. 8B**).

During development, Runx1 expression is required for the proper differentiation of Ret-expressing neurons (including those co-expressing GFRα2) from the early TrkA-positive population, that contains the majority of nociceptive neurons [29], [30], [31]. In the adult, Runx1 is expressed in the majority of Ret/IB_4_-positive neurons including 92% of the Mrgprd-positive neurons (a G protein-coupled receptor found in 75% of IB_4_-positive neurons [32]), 79% of transient receptor potential cation channel subfamily member M8 (TRPM8)-expressing neurons (a cation channel gated by low temperatures and menthol) and 15% of CGRP-expressing neurons [33], [34]. In Runx1 knockout mice, no cells are lost but the number of Ret-expressing neurons is decreased by over 50%. However, the percentage of IB_4_-positive neurons is normal, indicating that not all characteristics of the non-peptidergic afferent population are regulated by Runx1 [34]. To test whether Runx1 is required for GFRα2 expression, *in situ* hybridization(ISH) was performed in DRG from WT and Runx1 knockout mice. **Figure 8A** shows that in Runx1 knockout mice GFRα2 expression is greatly decreased, suggesting that GFRα2 is under control of Runx1 expression. We also examined expression of Runx1 and GFRα1, 2, 3 after saphenous nerve axotomy. We found that following axotomy, the decrease in Runx1 correlates with a decrease in GFRα2, but not GFRα1 or GFRα3 (**Fig. 8** ***B***), suggesting that Runx1 continues to control GFRα2 expression in adulthood. ISH confirmed that axotomized sciatic afferents (visualized by ATF3 staining) do not express Runx1 mRNA 5 d post-axotomy (**Fig. 8** ***C***), although Runx1 mRNA could be visualized in ATF3-negative neurons. These data suggest that Runx1 expression is required to maintain GFRα2 expression in the adult.

## Discussion

The results presented here confirm previous studies showing that sensory neuron populations defined by expression of GFRα1-3 respond differently following *in vivo* axotomy or dissociation *in vitro* (a procedure that includes peripheral and central axotomy). The present experiments extend those studies by demonstrating that the GFRα2 population is unique in that following injury *in vivo* and *in vitro*, these neurons rapidly down-regulate expression of GFRα2 and begin to express a phenotype typical of the GFRα3 population, including expression of functional TRPV1 receptors. Moreover, whereas *in vitro* phenotypic changes associated with injury can be prevented in some sensory populations by growth factors including NGF and ARTN, the changes seen in the GFRα2 population cannot be reversed by addition of NRTN. Interestingly, the loss of GFRα2 may be regulated by the transcription factor Runx1, as Runx1 is required for the developmental expression of GFRα2 and Runx1 is down-regulated *in vivo* following nerve transection.

In many ways, growth factors in the GDNF family have unique and complementary roles to those in the neurotrophin family (NGF, BDNF, NT-3 or NT-4/5). Although there is a small population of cells during early development that express Ret (these appear to become primarily low threshold mechanoreceptors [30], [35], [36]), the majority of GFL-responsive neurons initially express TrkA during early development and only begin to express Ret and GFRα receptors after the period of embryonic programmed cell death [30], [36]. Transgenic ablation of NGF, BDNF or NT-3 produces a significant reduction in the number of sensory neurons present in adult DRG and trigeminal ganglia. In contrast, genetic deletion of GFL or their receptors has a more mild effect on DRG and trigeminal neurons, probably due to the fact that expression of GFRα 1-3 and Ret is initiated in most neurons later in development [37], [38], [39], [40], [41], [42], after the period of programmed cell death when up to half of all embryonic sensory neurons undergo apoptosis [43]. *In vitro*, we observed a small (but significant) increase in neuronal survival of adult neurons upon addition of NGF, GDNF or ARTN but no effect for NRTN. These effects were expected given the phenotype of GFL or GFRα knockout mice. However, GFL have been reported to significantly increase the survival of embryonic and neonatal neurons *in vitro* [22]. The more dramatic survival effect of GFL on young sensory neurons *in vitro* versus that seen in knockout mice may reflect the response of young neurons to the injury sustained during dissociation; i.e., the process of culturing young neurons may initiate death programs that can be reversed by addition of growth factors. It should be noted that neuronal survival in the absence of growth factors is low for embryonic and neonatal sensory neurons (<20% for neurons younger than P15), whereas survival for adult neurons is >70% 4 d after culturing ([22], current results), again suggesting that neonatal neurons are more vulnerable to injury-induced cell death. Indeed, recent studies showed that BDNF is required for survival of neonatal nociceptors *in vivo* [41].

GFRα2 is not the only sensory neuron growth factor receptor that is decreased following injury; both p75 and TrkA are somewhat decreased following peripheral nerve lesion, whereas both TrkB and TrkC have been reported to increase [44], [45]. However, the decrease observed in TrkA and p75 can be blocked by exogenous NGF [45], whereas NRTN has no effect on GFRα2 expression, and thus, it seems as if GFRα2-expressing neurons are hard-wired to down-regulate the expression of this receptor and to lose the ability to respond to NRTN. One possible mechanism for this difference is that GFRα2 may continue in adulthood to be dependent on the expression of Runx1, which regulates its expression during development. Thus, when Runx1 is decreased following axotomy, GFRα2 is also decreased. During embryogenesis, TrkA is co-expressed with Runx1 and appears to be regulated by Runx1 [29], [31], [34], [46]. However, during late development Runx1 is down-regulated in TrkA-expressing neurons, presumably freeing TrkA from regulation by this transcription factor. The observed up-regulation of GFRα1 and GFRα3 indicate that either these receptors are not regulated by Runx1 in the adult or that Runx1 is acting as a repressor, an action it exerts during development ([8], [47], present results).

In culture, only NGF and ARTN, but not NRTN or GDNF, can block upregulation of ATF3. Interestingly, in mouse, 80% of neurons that express GFRα3 also express TrkA [14] so it is not clear if the ability to regulate ATF3 is specific to the population of cells expressing these two receptors or if downstream signaling is different when Ret interacts with GFRα3 compared to Ret activation in combination with GFRα1 or GFRα2. (However, it should be noted that a population of GFRα3-expressing neurons may not express Ret [19]). That this is not the case is suggested by the studies of Averill et al [16] that showed both NGF and GDNF could prevent the upregulation of ATF3 in adult rat *in vivo* when applied intrathecally for two weeks. These results indicate that long-term activation of Ret via GDNF can regulate ATF3. Why this does not happen *in vitro* during the short time course examined here could be due to numerous differences in the experimental paradigm including species, the effect of culturing and the time course of the two experiments.

ATF3 was uniformly upregulated in all injured neurons examined regardless of the changes in GFRα receptor expression *in vivo* or *in vitro*. This suggests the importance of ATF3 to regeneration [18]. GFRα2 is expressed in the majority of cutaneous afferents (ca. 70%; [48], [49]) and these correspond physiologically to polymodal C-fibers (CPM) [50], [51]. These fibers appear to regenerate at the same rate as other C-fibers [52] and thus, expression of GFRα2 does not appear to be a prerequisite for regeneration. Interestingly, analysis of GFRα2 knockout mice indicates that these neurons fail to innervate the epidermis in the absence of NRTN/GFRα2 signaling during early postnatal development [48].

Previous studies by Stucky and others indicate that GFRα2 expression is required for detection of noxious heat in dissociated IB_4_-binding neurons [26]. Because only a quarter of these neurons express TRPV1 mRNA and all have normal heat sensitivity in TRPV1^−/−^ mice [51], the majority of these neurons possess the ability to transduce heat stimuli independently of TRPV1. However, following axotomy, the data presented here indicate that some of these neurons begin to express TRPV1. This could theoretically explain the increased thermal sensitivity of C-fiber polymodal nociceptors to noxious thermal stimulation seen after regeneration [52]. That IB_4_-positive neurons are capable of expressing TRPV1 in culture following inflammation has been reported [28], although other laboratories have seen no change in cultured inflamed cutaneous afferents [53]. Thus, the ability of inflammation to induce *de novo* TRPV1 expression in cutaneous afferents remains controversial.

In summary, the studies conducted here indicate that GFRα2-expressing neurons are unique relative to other populations of sensory afferents identified based on growth factor receptor expression. GFRα2 is rapidly and dramatically down-regulated in response to injury *in vitro* and *in vivo* and these neurons appear to switch phenotype so that they can respond to a related growth factor (ARTN) and express TRPV1, a channel not normally seen in these neurons. These changes have obvious functional implications for the development of cutaneous hypersensitivity following injury. Identification of the signaling changes that lead to these alterations could provide new and useful therapeutic targets.

## Materials and Methods

### Animals

Experiments were conducted using young adult (6–8 weeks) male C57BL/6 mice (Jackson Lab). All animals were housed in group-cages, maintained on a 12 h light/dark cycle with a temperature-controlled environment, and given food and water *ad libitum*. All studies were performed in accordance with the guidelines of the Institutional Animal Care and Use Committee at the University of Pittsburgh and the National Institutes of Health *Guide for the Care and Use of Laboratory Animals*.

### Cell culture

Primary cultures were prepared as previously described [54]. Briefly, mice were given an overdose of avertin anesthetic and perfused transcardially with 4°C Ca^2+^/Mg^2+^ free Hank's balanced salt solution (HBSS). DRG were rapidly dissected and enzymatically treated with papain followed by collagenase to facilitate dissociation. DRG were then triturated in 0.5 ml F12 growth media (Invitrogen) containing 10% fetal calf serum and antibiotics (penicillin/streptomycin, 50 U/mL), and plated on laminin/poly-d-lysine coated dishes with a density of 1.85×10^5^. Plated cells were fed 2 h later with F12 growth medium containing 10% FCS (fetal calf serum) and antibiotics (penicillin/streptomycin, 50 U/ml). The growth factors were added to the medium at following concentrations: NGF (50 ng/ml), GDNF (50 ng/ml), ARTN (20 ng/ml), NRTN (50 ng/ml). For the experiment with application of both NRTN and GFRα2, three different sets of concentrations were used: NRTN (50 ng/ml), GFRα2 (100 ng/ml); NRTN (100 ng/ml), GFRα2 (200 ng/ml); NRTN (200 ng/ml), GFRα2 (400 ng/ml).

### Animal Surgery

All surgical procedures were performed under sterile conditions in a designated animal surgery area. Anesthesia was initiated by inhaled 4% isoflurane and maintained with inhaled 2% isoflurane. To assess changes in mRNA expression for GFRα1-3 and Runx1 induced by peripheral nerve axotomy, we performed sciatic and saphenous nerve axotomy. For sciatic nerve axotomy, the left hind leg was shaved, the skin was sterilized with betadine, the left sciatic nerve was exposed at the level of the head of the femur, transected and the wound was closed with wound clips. Saphenous nerve axotomy was performed on the right leg. A 5–6 mm incision was made in the skin at the mid-thigh level and the saphenous nerve was gently exposed and transected using fine scissors. Wounds were closed using 7.0 prolene sutures. At each time point studied, mice were given an overdose of avertin anesthetic, perfused with saline (followed in some cases by 4% paraformaldehyde) and DRG L4-5 (source of sciatic nerve primary afferents) from the left side or DRG L2-3 (source of saphenous nerve primary afferents) from the right side were collected.

To back-label non-peptidergic, IB_4_-binding, cutaneous afferents (the majority of which express GFRα2), 10 µl of 2 µg/µl IB_4_-488 was injected subcutaneously into dorsal medial side of both hindpaws for retrograde transport via the saphenous nerve to L2-3 DRG. Three days later, the saphenous nerve was transected. These mice were used to identify changes in gene and protein expression in the IB_4_-binding afferents following nerve transection. Calcium imaging and immunocytochemical staining and single cell RT-PCR were carried out 1–6 d after nerve transection.

To determine whether saphenous nerve axotomy had an effect on retrograde transport of IB_4_-488, we compared the percentage of neurons labeled by IB_4_-488 in two groups of mice. One group of mice had IB_4_-488 injected and L2-3 DRG were collected 9 d later. The second group had IB_4_-488 injected, followed by saphenous nerve axotomy 3 d later. 6 d following axotomy, L2-3 DRG were collected and analyzed for the percent labeled by IB_4_-488. There was no difference in the percent labeling, suggesting that axotomy had no effect on IB_4_-488 transport during the time points used for these studies. To determine if nerve cut *in vivo* had any effect on IB4-binding (i.e., if nerve transection decreased the expression of the ganglioside that bind the IB4 lectin), some mice were back-labeled with IB_4_-488 prior to nerve cut, then allowed to survive for 6 more days prior to culture and stained with fluorescently tagged IB_4_-647. All of the IB_4_-488-back-labeled cells were stained with IB_4_-647, indicating that despite being transected 6 d prior to culturing, all cells still expressed the gangliosides that bind IB4.

### Immunohistochemistry

For *in vitro* studies, coverslips containing dissociated cells were fixed in 4% paraformaldehyde for 10 min, washed in 0.1 M phosphate-buffered saline (PBS), and then incubated in blocking solution (2% normal horse serum, 0.2% Triton X-100 in PBS, pH 7.4) for 60 min. Coverslips were then incubated in primary antibodies diluted in blocking solution at 4°C overnight. Rabbit anti-TRPV1 (1∶1000 Neuromics), goat anti-GFRα1 (1∶500 R&D Biosystems), goat anti-GFRα2 (1∶500 R&D Biosystems), goat anti-GFRα3 (1∶100 R&D Biosystems), mouse anti-NeuN (1∶100 Chemicon), rabbit anti-CGRP (1∶1000 Chemicon), rabbit anti-ATF3 (1∶200 Santa Cruz Biotechnology), rabbit anti-PGP9.5 (1∶100, Ultraclone) were used. Binding of primary antibodies was visualized with donkey anti-rabbit, donkey anti-goat or donkey anti-mouse secondary antibodies conjugated to Cy3 or Cy2 (1∶1000; Jackson Immunoresearch). Coverslips were mounted in DPX on slides and photographed. Images were captured using Leica Application Suite (LAS) software and LEICA DM 4000B microscope.

For immunohistochemistry of IB_4_-488 back-labeled DRG, mice received an overdose of avertin anesthetic followed by transcardial perfusion with 4% paraformaldehyde. DRG were collected, cryoprotected in 30% sucrose, embedded in OCT mounting medium, cut at 20 µm on a cryostat and mounted on Superfrost microscope slides. Immunolabeling was performed as described above.

To perform double-labeling with CGRP and ATF3 antibodies which are generated from rabbit antiserum, we used Fab secondary antibodies as previously described [55]. Coverslips were incubated with blocking solution at room temperature for 1 hr, incubated in CGRP antibody at 4°C overnight, washed with PBS, then incubated with goat anti-rabbit Fab fragments (1∶50; Jackson Immunoresearch) at 4°C overnight. CGRP staining was visualized using donkey anti-goat Cy3 antibody. Coverslips were then incubated with ATF3 antibody at 4°C for overnight and ATF3 binding was visualized with donkey using anti-rabbit Cy2 antibody.

The percentage of GFRα-positive cells (expressed as a percent of the total number of NeuN-positive cells) was calculated in L4 DRG from three mice using systematic random sampling as described previously [56]. A total of 200 NeuN-positive cells per animal was assessed. Cellular profiles with a clearly defined nucleus with robust immunoreactivity (at least 5 standard deviations above background intensity) were considered positive. Images taken with one wavelength of fluorescence were scored and then overlaid with images of the second wavelength, allowing scoring of single- and double-labeled cells.

### Cell size distribution

NIH ImageJ was used to measure the area of neurons. DRG sections were labeled with antibodies to PGP9.5 (a pan-neuronal marker), GFRα1, GFRα2 or GFRα3 separately. DRG from three animals were analyzed and 200 positively-stained neurons for each marker from each animal were measured.

### Analysis of *in vitro* cell survival

To determine the amount of cell death over time in culture and the effect of growth factors on survival, neurons were plated on gridded, numbered coverslips. Eight squares from each coverslip were randomly selected and the number of neurons in each square was counted at 6 h, 1 d and 4 dafter plating. Neurons were grown in standard media with one of the following: NGF (50 ng/ml), GDNF (50 ng/ml), NRTN (50 ng/ml), ARTN (20 ng/ml) or without growth factor. Coverslips from six mice were analyzed for each condition. The cell number at 6 h in each condition was normalized as 100%. Data were analyzed using SigmaStat software. Significance was tested using a two-way ANOVA, and Dunnett's posthoc test.

### RNA isolation and real-time RT-PCR

RNeasy Mini kits (Qiagen) were used to isolate total mRNA. RNA (1 µg) was DNased (Invitrogen) to remove genomic DNA, and then reverse-transcribed using Superscript II reverse transcriptase (RT) (Invitrogen). Real-time RT-PCR was performed as described previously [56] to determine the extent of expression of growth factor receptors in sensory neurons after sciatic nerve lesion. SYBR Green-labeled PCR amplification was performed using an Applied Biosystems 5700 real-time thermal cycler (Foster City, CA) controlled by Prism 7000 SDS software (Applied Biosystems). All samples were run in triplicate, and control reactions were run without template as negative controls with every amplification run. The relative fluorescence of SYBR Green bound to double-stranded DNA was compared with a passive reference for every cycle. Threshold cycle (Ct) values, the cycle number in which SYBR Green fluorescence rose above background, were recorded as a measure of initial template concentration. Relative fold changes in RNA levels were calculated by the ΔΔCt method using GAPDH as a reference standard: Ct values from triplicate samples were averaged and then subtracted from the reference standard, yielding ΔCt. The difference between the ΔCt of the experimental and control groups were then calculated (ΔΔCt). The relative fold change was determined as 2^−ΔΔCt^. Statistical significance was determined by *t*-test. Primers optimized for real-time RT-PCR were designed using Oligo software (Molecular Biology Insights) and shown in **Table 5**. **Table 5** also showed the melting temperature for each primer. The annealing temperature was 60°C.

**Table 5:** Primers (sequences and melting temperatures) used for real-time RT-PCR assays.

Gene	Forward Primer (5′–3′)	Tm(°C)	Reverse Primer (5′–3′)	Tm(°C)
**GAPDH**	ATGTGTCCGTCGTGGATCTGA	58.1	ATGCCTGCTTCACCACCTTCTT	58.6
**GFRα1**	GTGTGCAGATGCTGTGGACTAG	57.6	TTCAGTGCTTCACACGCACTTG	58.5
**GFRα2**	TGACGGAGGGTGAGGAGTTCT	59.1	GAGAGGCGGGAGGTCACAG	58.7
**GFRα3**	CTTGGTGACTACGAGTTGGATGTC	57.7	AGATTCATTTTCCAGGGTTTGC	54
**Runx1**	TTTCAAGGTACTCCTGCCTGA	55.2	CAGTGAGAAGGACCAGAGACT	55.3

### Calcium imaging

Fourteen to seventeen hours after dissociation, cells were loaded with Ca^2+^ indicator by incubation in HBSS containing 5 mg/ml bovine serum albumin and 2 µM of the acetoxymethyl ester of fura-2 (Invitrogen) for 30 min at 37°C. Coverslips were placed on an Olympus microscope stage mount with 30°C HBSS buffer flowing at 5 ml/min. Firmly attached cells with IB_4_-488 labeling were chosen and identified as regions of interest in the software (Simple PCI; C-Imaging, Compix Imaging Systems). Emission data at 340 and 380 nm were collected at 1 Hz, and the change in the 340/380 ratio analyzed. Ca^2+^ transients were examined in response to brief application of 1 µM capsaicin (Sigma) delivered onto neurons using a multi-barrel drug delivery system.

### *In situ* hybridization

The protocol for GFRα2 ISH was reported previously [34]. Briefly, an ISH probe for GFRα2 was amplified using a nested PCR strategy with gene-specific sets of PCR primers and cDNA templates prepared from P0 mouse whole brain. The probe was labeled with digoxigenin (Roche). To test the dependence of GFRα2 expression on the presence of functional Runx1, P30 *Runx1^F/F^* and *Runx1^F/F^;Wnt1-cre* mice were perfused with 4% paraformaldehyde. Lumbar DRG were dissected, post-fixed for 1–2 h, cryoprotected overnight in 20% sucrose and cut at a thickness of 12 µm. Slides containing tissue sections with both genotypes were pretreated with proteinase K and TEA/acetic anhydride before being incubated overnight at 64°C with the GFRα2 probe. Reaction with the NBT/BCIP substrate (Roche) was allowed to proceed overnight. For experiments in which Runx1 ISH was combined with ATF3 immunolabeling, L3-5 DRGs were collected 5 d after sciatic axotomy (see above). Ganglia were first stained with anti- antibody (rabbit 1∶1000, Santa Cruz) and photographed, followed by development for Runx1 ISH using fluorescently-tagged tagged nucleotides [34].

### Single Cell RT-PCR

All mice were injected in footpad skin with IB_4_-488 as described above (9 d prior to sacrifice). Two to four hours after dissociation and culture, IB_4_-back-labeled DRG neurons were collected under fluorescence microscope (Leica) with large-bore (∼50 µm) glass pipettes and expelled into microcentrifuge tubes containing reverse transcriptase mix (Invitrogen). For each experiment, negative controls consisted of omitting RT or using a cell-free bath aspirate as template. The first-strand cDNA was used as template in a PCR reaction containing 1×GoTaq reaction buffer (Promega); primer sequences are listed in **Table 6**. Each initial PCR product served as template in a subsequent PCR using a nested or semi-nested primer pair, the products of which were electrophoresed on 2% agorose-ethidium bromide gels and photographed. Only samples producing detectable amplification of positive control housekeeping gene (GAPDH) were analyzed.

**Table 6:** Primers used for single cell RT-PCR.

Gene (expected size)	External primers	Internal primers
ATF3 215 bp, 99 bp	ACCTCCTGGGTCACTGGTATTTC TTCTTTCTCGCCGCCTCCTTTTCC	AGTCAGTTACCGTCAACAACAGACC TTCTTTCTCGCCGCCTCCTTTTCC
GFRα1 223 bp, 219 bp	GTGCTCCTATGAAGAACGAGAGAG GCTGCTGGAGTCTATGTAGTTAGG	TCCTATGAAGAACGAGAGAGGC GCTGCTGGAGTCTATGTAGTTAGG
GFRα2 298 bp, 260 bp	CTAGTAGGACAAAGAGAAGCCC GTCCTTGAGGAACTTCTCACACTC	CTGTGTCGTACAGACCACTTGT GTCCTTGAGGAACTTCTCACACTC
GFRα3 599 bp, 279 bp	GCCCAGGCTACCCATTCTTTCTTTCT CAAATGTTCAGGATTGCCTGGCAGAG	CTCCTTAGGACTTTGTGGGTCCAGTT CAAATGTTCAGGATTGCCTGGCAGAG
TRPV1 486 bp, 191 bp	GGGAAGAATAACTCACTGCCTGTG TGGGTCCTCGTTGATGATGC	GGCGAGACTGTCAACAAGATTG TCATCCACCCTGAAGCACCAC

### Statistical analysis

All the detail of statistical analysis is listed at the end of figure and table legends.
